# Mobile application for first aid to children: a technological innovation in schools

**DOI:** 10.1590/0034-7167-2024-0057

**Published:** 2025-03-10

**Authors:** Rosana Castelo Branco de Santana, Márcia Maria Carneiro Oliveira, Amanda Aparecida Silva Rios, Nelson Miguel Galindo

**Affiliations:** I Universidade Federal da Bahia. Salvador, Bahia, Brazil; II Instituto Federal de Educação, Ciência e Tecnologia de Pernambuco. Pesqueira, Pernambuco, Brazil

**Keywords:** Mobile Applications, First Aid, Accidents, Child Health, Nursing Informatics, Aplicaciones Móviles, Primeros Auxilios, Accidentes, Salud Infantil, Informática Aplicada a la Enfermería

## Abstract

**Objectives:**

to develop a mobile application for first aid to children, designed for use by basic education professionals.

**Methods:**

we carried out this applied research in three phases: 1-integrative review, 2- identification of learning needs through a cross-sectional study with 53 school professionals, and 3- app development.

**Results:**

the Child and Care (*Criança e Cuidado*) app includes three main sections (Important contacts, Learn first aid, and Record the accident). It incorporates multimedia resources such as videos and images to inform users about first aid in the school environment and allows accident logging.

**Conclusions:**

this mobile app is a technological educational tool that, after validation, will disseminate crucial information to protect children’s health and safety. It is an initiative to prevent unintentional injuries and provide a strategy for expanding the role of nurses by integrating health and education.

## RESUMEN

**Objetivos:**

desarrollar una innovación tecnológica en formato de aplicación móvil sobre primeros auxilios para niños, destinada a profesionales de la educación básica.

**Métodos:**

investigación aplicada para producción tecnológica en tres fases: 1-revisión integrativa, 2-identificación de necesidades de aprendizaje (mediante estudio transversal con 53 profesionales escolares) y 3-construcción de la aplicación.

**Resultados:**

la aplicación “Niños y Cuidados” consta de 3 *moblets* principales (Contactos importantes; Aprenda primeros auxilios; Registre aquí el accidente), incluye recursos como vídeos e imágenes, y tiene como objetivo informar sobre primeros auxilios a niños en el entorno escolar, permitiendo registrar accidentes ocurridos.

**Conclusiones:**

la aplicación móvil es una herramienta educativa tecnológica que, tras validarse, permitirá divulgar información esencial para la protección de la vida y salud de los niños. Representa una iniciativa para enfrentar lesiones no intencionadas infantiles y una estrategia para ampliar el ámbito de actuación de las enfermeras al articular salud y educación.

## INTRODUCTION

The high incidence of childhood accidents, their impact on quality of life, and mortality rates emphasize the need for strategies to address unintentional injuries among children. The epidemiological scenario, children’s immaturity in recognizing risky situations, and their natural curiosity during development necessitate constant attention to ensure a healthier life for this population.

In 2019, the World Health Organization (WHO) began developing the Global Strategy on Digital Health, which aims to enhance national efforts through the creation and exchange of knowledge to promote health for all across different regions^(1)^. Children tend to spend a considerable amount of time in the school environment, which is conducive to accidents due to the large number of children present. At the same time, it serves as an ideal space for health promotion and education initiatives.

Among health interventions, school first aid actions are particularly important as teachers and other professionals must know how to respond to health incidents involving children, aiming to minimize potential long-term effects and safeguard lives^(2)^. While limited in Brazilian society, this knowledge must be disseminated and expanded. Thus, the integration between the health and education sectors is a key component of Brazil’s School Health Program, which nurses can support. In this regard, a study identified accidents as one of the primary causes of child hospitalization and concluded that nursing could develop strategies as outreach programs focused on safety measures and prevention^(2)^.

However, another study highlighted teachers’ limited knowledge of first aid practices^(2)^. Therefore, training basic education professionals in using mobile applications is essential. The advancement of Information and Communication Technologies (ICT) and the versatility of mobile devices demonstrate that these tools are commonplace in daily life and significantly influence the learning process in both the health and education sectors^(4)^. However, many professionals are not fully prepared to adopt these technologies, as shown in a review that analyzed health applications and revealed a gap in first aid-related resources^(5)^. These findings justify the development of this study.

## OBJECTIVES

To develop a technological innovation in the form of a mobile application on first aid for children, designed for use by basic education professionals.

## METHODS

We conducted a technological production study to develop a mobile application following the criteria recommended by Apple’s Human Interface Guidelines^(6)^. The study consisted of three phases: integrative review, identification of learning needs, and mobile application development. This research was carried out from October 2021 to August 2023.

In the first phase, we performed an integrative literature review to identify the main types of school accidents affecting children. The research problem was framed using the PICo acronym (Population, Interest, Context): Population – pediatric; Phenomenon of Interest – accidents; and Context – school environment. The research question was formulated as follows: What scientific evidence exists regarding accidents in school environments involving pediatric populations?

We searched on PubMed, Embase, Virtual Health Library (VHL), and CINAHL, combining the descriptors Pediatrics (or Child), Accidents (or Accident Prevention), First Aid, and Schools (or “Child Day Care Centers” or “Nurseries, Infant” or “School Health Services”). Thus, we excluded non-primary articles, such as literature reviews, opinion pieces, letters to the editor, essays, preliminary notes and manuals, monographs, dissertations, theses, duplicates, and those not addressing the research question. The articles underwent a comprehensive screening process, with each article independently and blindly reviewed by the authors to critically assess its relevance to the study’s objectives and its alignment with inclusion criteria. In cases of disagreement, a third author reviewed and determined the final inclusion or exclusion. Ultimately, seven articles were selected for the sample.

In the second phase, we focused on planning the app by addressing the learning needs identified by school professionals. For this stage, we conducted interviews with primary education professionals from a capital city in the Northeast region of Brazil, using the following key question:”lf an app providing guidance on first aid were offered, what topics would you like to learn about?” The interviews were conducted after obtaining approval from the Research Ethics Committee, and the data were processed using NVIVO software. Based on each identified need, we gathered available scientific evidence on appropriate first aid procedures, referencing guidelines from the American Heart Association (AHA) and the Brazilian Society of Aquatic Rescue.

In the third phase, the app development stage, which included both Design and Implementation, we converted the system specification into an executable system, creating the functional app for user access on their devices. A technical team, including a graphic designer, a software engineer, and researchers, carried out this phase. We used the User-Centered Design (UCD) approach. This method fosters collaboration between users and designers/ researchers in the initial design phase to develop digital systems, such as mobile applications. During the app’s UCD process, users contributed to its design by providing input on their needs and participating in usability testing.

We implemented specific software development techniques throughout the app’s development, including the Unified Modeling Language (UML) and the creation of diagrams, such as use cases, class, and sequence diagrams. We also modeled the database, creating an Entity-Relationship Diagram and a data dictionary. We conducted a study on web application development and defined React.js for the front end, Node.js for the back end, and a database management system for building and maintaining the database. Additionally, we created system prototypes to gather preliminary feedback from health experts and users about the application. We also developed test plans and executed predefined scenarios, focusing particularly on ensuring the system requirements were correctly implemented and well-received by users.

## RESULTS

In the first phase, the integrative review identified seven observational studies (cross-sectional or longitudinal), six of which were conducted in developing countries. The most frequent school-related accidents among children were head trauma (5), fractures (5), burns (4), contusions (4), and sprains (4). In the second phase, 53 basic education professionals were interviewed to assess learning needs. Over 50% expressed interest in first aid for choking and convulsive crises. However, the professionals mentioned all types of accidents included in the app.

We named the app Child and Care to reflect its objectives: providing information on first aid for children in the school environment and enabling the recording of accidents occurring in this setting. In adherence to ethical guidelines for human subjects research, the research team intends to continue supporting children who experience accidents by monitoring their referrals and outcomes.

**Figure 1:**
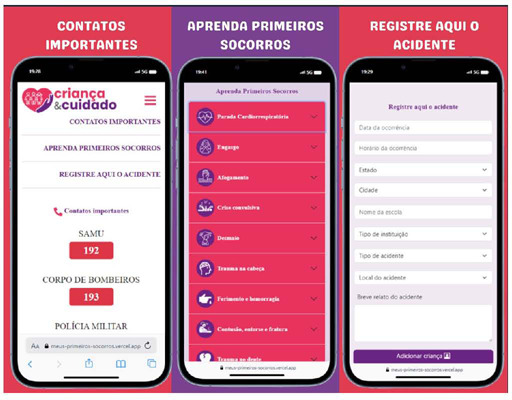
Main app modules: Important contacts, Learn first aid, and Record an accident

The app includes three main functionalities (moblets). The first presents essential emergency contacts (Mobile Emergency Care Service - SAMU – 192, Fire Department – 193, and Police Department – 190). The second moblet is titled “Learn first aid”, and the third is “Record the accident” (Figure 1).

In the “Learn first aid” section, accident types are described according to their occurrence in school settings: Cardiac arrest (CA), Choking, Drowning, Convulsive crisis, Fainting, Head trauma, Wound and bleeding, Contusion, Sprain, Fracture, Tooth trauma, and Burn.

The”Record the accident” section is designed to enable notifications of any accidents occurring within the school environment. It is intended for school staff to complete. It includes fields for details such as the accident’s date, time, state, city, the child’s name, the type of educational institution (public or private), the type of accident (with options covering various incident types), and the specific location within the school where the accident happened. Each item provides an “other” option to allow staff to document any circumstances not covered by the existing selections, followed by a text box for a subjective description of the accident. After completing the form, staff members can click “add child” to submit the report. The lead researcher will securely store all recorded information to monitor the child’s follow-up care. Following app validation, it is important to note that child registration and monitoring will only be conducted with the local ethics committee’s approval and after obtaining signed informed consent from the child’s parents or guardians.

**Figure 2:**
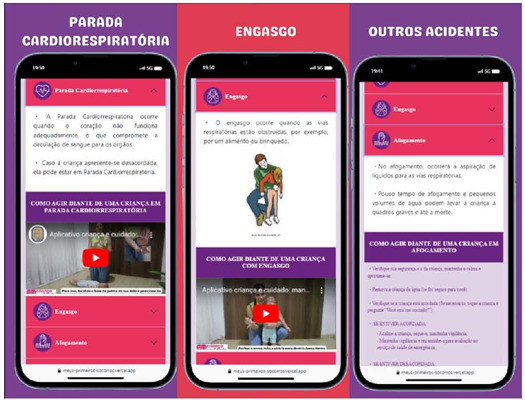
Screen of the “Learn first aid” moblet with first aid definitions and steps

As shown in Figure 2, in the “Learn first aid” section, selecting each accident type opens a new page with information on 1) the definition of the accident and 2) appropriate response measures for aiding an injured child. Information on item 1 for CA and choking is presented through videos recorded by the study’s primary author, while items 1 and 2 for other accidents are detailed in text format.

## DISCUSSION

The app’s homepage presents “Important contacts” for accessing emergency first aid services. According to the AHA, activating the emergency medical service is essential in the pediatric out-of-hospital CA survival chain^(7)^. An observational study involving 27,705 patients showed that individuals who had out-of-hospital CA and were transported by trained teams had better outcomes compared to continuous on-site resuscitation efforts^(8)^. This emphasizes the importance of promptly contacting pre-hospital care teams. However, evidence points to a gap between ideal practice and reality. A Brazilian study identified that most laypeople do not know the correct number for activating the Mobile Emergency Care Service (SAMU)^(9)^.

The second moblet, “Learn first aid”, addresses the most common accidents in school settings. According to the WHO, accidents affect children globally, negatively affecting families and society. These incidents occur in various environments frequented by children, particularly in schools, which should serve as spaces for health education. Given the repercussions of childhood accidents, prevention becomes crucial. In line with this, a study by Sleet^(10)^ identified the global challenge of preventing childhood injuries and advocated for prevention programs through policies, strategies, and action plans.

The articulation between health and education sectors is the School Health Program’s foundation, vital for promoting child health. Nurses play a crucial role in addressing accidents involving children, bringing benefits to school health promotion. They can engage in actions ranging from identifying injury risks to providing first aid guidance, helping to create safer school environments by improving survival rates and reducing long-term sequelae.

Like other professions, nursing must align with digital technology, which permeates various aspects of daily life. Moreover, digital interventions positively impact health behavior changes in different contexts^(11)^. This study could contribute significantly to nursing by discussing health education in schools, thus opening a new area of practice and reinforcing the connection between health and education through digital health.

The third moblet, “Record the accident,” allows users to notify and document accidents. Mobile technology for health data collection offers several benefits, such as improved information quality, data storage, easier information processing, and enhanced security in result analysis^(12)^. Continuous monitoring of childhood accidents could reduce underreporting and facilitate the diagnosis of factors leading to accidents, enabling the implementation of effective intervention strategies in schools.

It is important to note that the Child and Care mobile app has an educational purpose; hence, it is not intended for immediate use during an accident. For initial emergency response, school staff should know how to respond to emergencies based on prior training and app usage, which serves to clarify doubts. The app may also be used to record accidents after the child has been referred for care.

This technological innovation can disseminate first aid knowledge for children beyond schools, contributing to child health improvement across national settings. The app could be used as an educational tool in first aid courses and may be extended to families, as accidents also occur outside schools, particularly at home. As evidence of the gap that apps can address, a study in Portugal involving families of children aged 5 to 9 identified a lack of knowledge about caring for injuries and burns^(13)^.

Our app aims to protect children’s lives and minimize sequelae from inadequate first aid; advance scientific knowledge and drive social transformation in children’s lives; and reinforce the National Policy for Comprehensive Child Health Care and the School Health Program. Additionally, it aligns with Brazil’s 2020-2028 Digital Health Strategy, designed to guide and align activities focused on expanding and transforming digital health in the country.

### Study limitations

This study was limited by the scarcity of available information sources for the integrative review. Additionally, the app lacks accessibility features for the deaf and mute communities, limiting its compliance with inclusive digital health standards.

### Contributions to the fields of science and nursing

Regarding the implications for advancing scientific knowledge in health and nursing, the Child and Care app, once validated, will be made publicly available, aiming to reach as many Brazilian schools as possible and to be used by nurses in health education initiatives. Furthermore, the app could be a management tool for health and education departments.

## CONCLUSIONS

We developed a technological innovation in the form of a mobile app for first aid care for children, designed for use by basic education professionals. The app allows for recording accidents within school environments. It is structured around three main moblets: Important contacts, Learn first aid, and Record the accident. The app includes resources such as videos, images, and text information organized into clear topics.

This app is a technological educational tool that, after validation, will enable the widespread dissemination of essential information to protect children’s health and lives. Thus, it represents a significant initiative to combat unintentional childhood injuries, which remain a major public health concern and a critical factor in child morbidity and mortality.
