# Supplementary material for: Mobile application for first aid to children: a technological innovation in schools
Source: Rev Bras Enferm. 2025 Mar 10;78(1):e20240057. doi: 10.1590/0034-7167-2024-0057 (PMC11893059; doi:10.1590/0034-7167-2024-0057)
Supplement: Translation

# Translation: Aplicativo móvel sobre primeiros socorros a crianças: inovação tecnológica nas escolas

**DOI:** 10.1590/0034-7167-2024-0057pt

**Authors:** Rosana Castelo Branco de Santana, Márcia Maria Carneiro Oliveira, Amanda Aparecida Silva Rios, Nelson Miguel Galindo

**Affiliations:** I Universidade Federal da Bahia. Salvador, Bahia, Brasil; II Instituto Federal de Educação, Ciência e Tecnologia de Pernambuco. Pesqueira, Pernambuco, Brasil

**Keywords:** Aplicativos Móveis, Primeiros Socorros, Acidentes, Saúde da Criança, Informática em Enfermagem

## Abstract

**Objetivos:**

desenvolver inovação tecnológica no formato de aplicativo móvel sobre primeiros socorros a crianças, para uso por profissionais da educação básica.

**Métodos:**

pesquisa aplicada de produção tecnológica, composta por três fases: 1 -revisão integrativa, 2-identificação das necessidades de aprendizagem (mediante pesquisa transversal com 53 profissionais de escolas) e 3-construção do aplicativo.

**Resultados:**

o aplicativo Criança e Cuidado é composto por 3 *moblets* principais (Contatos importantes; Aprenda primeiros socorros; e Registre aqui o acidente), contém recursos como vídeos e imagens e objetiva informar sobre primeiros socorros a crianças no ambiente escolar, possibilitando o registro dos acidentes ocorridos.

**Conclusões:**

o aplicativo móvel é uma ferramenta educacional tecnológica que, após validação, permitirá a divulgação de informações essenciais para proteger a vida e saúde das crianças. Por isso, representa iniciativa de enfrentamento às lesões não intencionais infantis e estratégia para ampliar o espaço de atuação de enfermeiras ao articular saúde e educação.

## INTRODUÇÃO

A alta incidência de acidentes com crianças, as suas repercussões para a qualidade de vida e o perfil de mortalidade remetem à importância de estratégias de atuação ante as lesões não intencionais infantis. O cenário epidemiológico, a imaturidade das crianças para identificar situações arriscadas do ambiente e a curiosidade inerente ao desenvolvimento humano nessa faixa etária exigem olhares permanentes para encontrar caminhos que assegurem ao público infantil uma vida mais saudável.

Em 2019, a Organização Mundial da Saúde (OMS) iniciou a elaboração da *Global Strategy on Digital Health,* que é a estratégia global de saúde digital, cuja compreensão é de que os esforços nacionais devem ser potencializados pela elaboração e troca de conhecimento relacionado à promoção da saúde para todos em diversos lugares^(1)^. O ambiente escolar é onde crianças tendem a passar considerável parte do tempo, sendo um lugar propício aos acidentes devido ao grande número de crianças ali, representando, ao mesmo tempo, um cenário privilegiado para promoção e educação em saúde.

Dentre as práticas para a saúde, destacam-se as ações de primeiros socorros no ambiente escolar, pois é preciso que professores e demais profissionais saibam agir diante de situações de intercorrências de saúde às crianças, visando contribuir para minimizar sequelas e preservar a vida^(2)^. Esse conhecimento, pouco difundido na sociedade brasileira, necessita ser estimulado e multiplicado. Assim, a articulação entre os setores saúde e educação é um componente-chave do Programa Saúde na Escola, que pode ser desenvolvido por enfermeiros. Nesse sentido, um estudo identificou os acidentes como uma das principais causas de internamento de crianças e concluiu que a enfermagem poderá desenvolver estratégias como programas de extensão com foco em medidas de segurança e prevenção^(3)^.

Entretanto, pesquisa identificou conhecimento limitado de professores para realizar primeiros socorros^(2)^. Por isso, é necessário capacitar os profissionais que trabalham na educação básica para o uso de aplicativos móveis, pois o avanço das Tecnologias da Informação e Comunicação (TIC) e a versatilidade do aparelho móvel mostram que esses dispositivos são ferramentas comuns no cotidiano e exercem influência significativa no processo de aprendizagem, inclusive nos setores saúde e educação^(4)^. Todavia, muitos profissionais ainda não estão plenamente preparados para adotar essas tecnologias, como aponta uma revisão que elencou os aplicativos na saúde e mostrou lacuna na temática de primeiros socorros^(5)^. São evidências como esta que justificam o desenvolvimento do presente estudo.

## OBJETIVOS

Desenvolver inovação tecnológica no formato de aplicativo móvel sobre primeiros socorros a crianças, criado para uso por profissionais da educação básica.

## MÉTODOS

Estudo de produção tecnológica para construir um aplicativo móvel baseado nos critérios recomendados pela Apple conforme descrição nas *Human Interface Guidelines* ^(6)^, em três fases: revisão integrativa, identificação das necessidades de aprendizagem e desenvolvimento de um aplicativo móvel. Foi realizado entre outubro de 2021 e agosto de 2023.

Na primeira fase, procedeu-se a uma revisão integrativa da literatura para identificar os principais acidentes que acometem crianças nas escolas. Na sequência, identificou-se o problema de pesquisa mediante o acrônimo PICo (População, Interesse, Contexto): População – pediátrica; Fenômeno de interesse – acidentes; e Contexto – ambiente escolar. Assim, estabeleceu-se a seguinte questão de pesquisa: Quais as evidências científicas sobre acidentes no ambiente escolar com a população pediátrica?

Na ocasião, efetuou-se busca na PubMed (National Library of Medicine), Embase, Biblioteca Virtual de Saúde (BVS) e Cumulative Index to Nursing and Allied Health Literature (CINAHL) por meio da combinação dos descritores *Pediatrics* (ou *Child*), *Accidents* (ou *Accident Prevention*), *First Aid* e *Schools* (ou *“Child Day Care Centers”* ou *“Nurseries, Infant”* ou *“School Health Services”*). Assim, foram excluídos artigos não primários como as revisões de literatura, artigos de opinião, cartas ao editor, ensaios, notas prévias e manuais, monografias, dissertações, teses, duplicados e os que não respondessem à questão da pesquisa. A triagem dos artigos ocorreu após leitura na íntegra de forma independente e às cegas pelos autores para análise crítica em relação à sua aderência ao objeto desta pesquisa e critérios de inclusão. Em caso de divergências, uma terceira autora fez a leitura e decidiu pela inclusão ou exclusão. Ao final, sete artigos foram incluídos na amostra.

Na segunda fase, para o planejamento do aplicativo, foram consideradas as necessidades de aprendizagem apontadas por profissionais de escolas. Para essa etapa, foram feitas entrevistas com profissionais da educação básica de uma capital do Nordeste, com a seguinte pergunta-chave: “Se fosse ofertado um aplicativo com orientações sobre primeiros socorros, quais assuntos você gostaria de aprender?” As entrevistas ocorreram após aprovação do Comitê de Ética em Pesquisa e os dados foram processados pelo software NVIVO. Assim, para cada demanda identificada, foram levantadas as evidências científicas disponíveis sobre a adequada conduta de primeiros socorros com base na American Heart Association (AHA) e Sociedade Brasileira de Salvamento Aquático.

Na terceira fase, a de desenvolvimento do aplicativo, que inclui o Projeto e Implementação, deu-se a conversão da Especificação do sistema em um sistema executável, ou seja, o aplicativo funcional, que pode ser utilizado pelos usuários em seus dispositivos. Essa etapa foi desenvolvida pela equipe técnica, composta por um profissional de design gráfico, um engenheiro de software e pesquisadores. Foi usado o método Design Centrado no Usuário (DCU) - *User Centered Design.* Trata-se de um método que estabelece a participação/colaboração entre os usuários e os designers/pesquisadores na fase da concepção para desenvolver sistemas informatizados, como os aplicativos móveis. No DCU do aplicativo, os usuários participaram da concepção do *app,* sendo consultados sobre suas necessidades e convidados a participar do teste de usabilidade.

Durante o desenvolvimento do aplicativo, foi feita a modelagem do sistema por meio de técnicas específicas para desenvolvimento de software, o que incluiu a utilização da UML (*Unified Modeling Language*) e a construção de diagramas, como de casos de uso, classes e sequência; Modelagem do banco de dados, incluindo a construção do diagrama Entidade-Relacionamento e o dicionário de dados; e Estudo de desenvolvimento de aplicações web e definição da linguagem *React.js* utilizadas no *front-end,* da linguagem *Node.js* usada no *back-end* e sistema de gerenciador de banco de dados para a criação e manutenção da base de dados. Além disso, foi feita a Prototipação do sistema de maneira a antecipar o feedback dos especialistas em saúde e usuários sobre a aplicação; e a Criação de planos de testes e execução dos cenários estabelecidos, especialmente aqueles relacionados à correta execução dos requisitos do sistema e boa aceitação pelo usuário.

## RESULTADOS

Na primeira fase do estudo, a revisão integrativa encontrou sete artigos, todos observacionais (transversais ou longitudinais), dos quais seis foram realizados em países em desenvolvimento. Os acidentes infantis com maior frequência no ambiente escolar foram: trauma na cabeça (5), fratura (5), queimadura (4), contusão (4) e entorse (4). Na segunda fase, para identificar as necessidades de aprendizagem, foram entrevistados 53 profissionais de educação básica. Mais de 50% dos profissionais relataram interesse por primeiros socorros em situações de engasgo e crise convulsiva. Porém, considerando a totalidade dos relatos, foram mencionados todos os tipos de acidente contidos no aplicativo.

O aplicativo foi intitulado Criança e Cuidado, pois seus objetivos são: informar sobre primeiros socorros a crianças no ambiente escolar e possibilitar o registro dos acidentes ocorridos nesse cenário. Respeitando os preceitos éticos da pesquisa com seres humanos, a equipe de pesquisa pretende continuar a cuidar das crianças vítimas dos acidentes, monitorando o encaminhamento e o desfecho.

O aplicativo é composto por três *moblets* (funcionalidades/ambientes do *app*). O primeiro contém uma tela inicial com contatos importantes dos serviços de primeiros socorros (Serviço de Atendimento Móvel de Urgência – SAMU – 192; Corpo de Bombeiros – 193; e Polícia Militar - 190). Os outros dois *moblets* são “Aprenda primeiros socorros” e “Registre aqui o acidente” (Figura 1).

**Figura 1:** Moblets principais: Contatos importantes, Aprenda primeiros socorros e Registre aqui o acidente

No *moblet* “Aprenda primeiros socorros,” descrevem-se os tipos de acidentes conforme sua ocorrência no ambiente escolar: Parada cardiorrespiratória (PCR); Engasgos; Afogamento; Crise convulsiva; Desmaio, Trauma na cabeça; Ferimento e hemorragia; Contusão, entorse e fratura; Trauma no dente; e Queimadura.

O *moblet* “Registre aqui o acidente” objetiva possibilitar a notificação dos acidentes ocorridos. Deve ser preenchido por um funcionário da escola e contém as seguintes informações do acidente (ocorrência): data, horário, estado do Brasil, cidade do Brasil, nome da criança, tipo de instituição de ensino (pública ou privada), tipo do acidente (traz a opção de todos os acidentes) e local do acidente dentro da escola (traz a opção dos diversos espaços do ambiente escolar). Em todos os itens, existe a opção “outro” para que o funcionário possa descrever uma situação não disponível nas opções anteriores; e, ao final, há uma caixa de texto para descrição subjetiva do acidente. Após preenchimento, clica-se no botão “adicionar criança”. Todas essas informações serão arquivadas pela pesquisadora principal para monitorar essa criança. Vale ressaltar que, depois da validação do aplicativo, será realizado o cadastro e acompanhamento das crianças apenas após aprovação pelo comitê de ética do local onde o *app* estiver instalado e assinatura do Termo de Consentimento Livre e Esclarecido pelos pais ou responsáveis pela criança.

Conforme Figura 2, no *moblet* “Aprenda primeiros socorros”, a seleção de cada tipo de acidente permite a abertura de novo *moblet* com informações sobre: 1) Definição do acidente e 2) Como agir diante de uma criança acidentada. As informações do item 1 para as situações de PCR e engasgo foram apresentadas em vídeos gravados pela autora principal do estudo. Para os demais acidentes, as informações 1 e 2 estão descritas em texto.

**Figura 2:** Tela do moblet “Aprenda primeiros socorros” com as definições e condutas de primeiros socorros

## DISCUSSÃO

Em sua página inicial, o aplicativo apresenta os “Contatos importantes” para o acionar os serviços de primeiros socorros. Segundo recomendações da AHA, na cadeia de sobrevivência da PCR extra-hospitalar pediátrica, é preciso acionar o serviço médico de emergência^(7)^. Estudo observacional com 27.705 pacientes identificou que pacientes que tiveram PCR extra-hospitalar e foram transportados por equipe treinada tiveram alta hospitalar com desfecho favorável, quando comparados com os que receberam reanimação contínua no local do acidente^(8)^. Isso reforça a importância do acionamento imediato da equipe de assistência pré-hospitalar. Contudo, evidências expõem uma distância entre o ideal e o real. Estudo brasileiro identificou que a maioria dos leigos desconhecem o número correto para acionamento do Serviço de Atendimento Móvel de Urgência (SAMU)^(9)^.

O segundo *moblet,* “Aprenda primeiros socorros”, apresenta os principais acidentes que ocorrem no ambiente escolar. Segundo a OMS, os acidentes atingem crianças em todo o mundo e trazem impactos negativos para família e sociedade. Podem ocorrer nos diferentes cenários por onde as crianças transitam, principalmente nas escolas, que, por sinal, devem ser espaços promotores de educação para saúde. Diante das repercussões causadas por acidentes na infância, enfatiza-se a necessidade de preveni-los. Em consonância, um estudo realizado por Sleet^(10)^ identificou que prevenir lesões infantis é um desafio global e defendeu a necessidade de programas de prevenção mediante políticas, estratégias e planos de ação.

Assim, ressalta-se a importância da articulação entre os setores saúde e educação, base do Programa Saúde na Escola, em prol da saúde infantil. Nesse sentido, enfermeiros podem ocupar papel fundamental no enfrentamento dos acidentes com crianças, trazendo benefícios para a promoção da saúde escolar. Eles podem atuar em diferentes ações, desde a identificação de riscos para as lesões até a orientação sobre os primeiros socorros, o que pode contribuir para tornar o ambiente escolar mais seguro por meio do aumento da sobrevida e da redução de sequelas.

O exercício da enfermagem, assim como de outras profissões, necessita de uma atuação harmonizada com a tecnologia digital, a qual está presente em diferentes dimensões da vida cotidiana. Além disso, as intervenções digitais impactam positivamente e promovem mudança de comportamento em relação à saúde em diferentes contextos^(11)^. É relevante a contribuição que o estudo poderá representar para a Enfermagem ao discutir a educação em saúde no ambiente escolar, aproximando-se de mais um campo de ação para a profissão e, assim, fortalecendo a relação educação-saúde por meio da saúde digital.

O terceiro *moblet* do aplicativo, “Registre aqui o acidente”, traz espaços para notificação dos acidentes ocorridos. Há diversos benefícios no uso da tecnologia móvel para coleta de dados em saúde, como qualidade na informação, auxílio e armazenamento de dados, facilidade no processamento das informações e maior segurança na análise dos resultados^(12)^. O monitoramento contínuo dos acidentes infantis pode reduzir as subnotificações e facilitar o diagnóstico de problemas que levam à ocorrência dos acidentes, com a consequente implementação de estratégias de intervenção efetivas no ambiente escolar.

Vale ressaltar que o aplicativo móvel Criança e Cuidado tem proposta educativa, logo não é recomendado para uso no momento do acidente. Para o atendimento inicial nas situações de emergência, os profissionais das escolas devem saber como agir com os conhecimentos adquiridos previamente por periódicas capacitações e uso anterior do aplicativo, sanando dúvidas. Esse *app* pode ser utilizado ainda para o registro do acidente após o encaminhamento da criança.

A inovação tecnológica do aplicativo móvel pode disseminar o conhecimento sobre primeiros socorros em crianças para além do ambiente escolar e contribuir para o fortalecimento da saúde da criança em diferentes cenários nacionais. O aplicativo pode ser usado como ferramenta educacional em cursos de primeiros socorros, e seu uso pode ser estendido para familiares, uma vez que os acidentes também acontecem fora do ambiente escolar, com destaque para acidentes domésticos. Como evidência da lacuna que os aplicativos podem preencher, estudo realizado em Portugal com familiares de crianças de 5 a 9 anos identificou falta de conhecimento acerca dos cuidados com ferimentos e queimaduras^(13)^.

Nosso aplicativo visa proteger a vida e reduzir sequelas nas crianças decorrentes de primeiros socorros inadequados; fortalecer o avanço científico e transformação social na vida das crianças; e consolidar a Política Nacional de Atenção Integral à saúde da criança e o Programa Saúde na Escola. Adicionalmente, pretende contribuir para as Estratégias de Saúde Digital para o Brasil 2020-2028, cujo objetivo é nortear e alinhar atividades voltadas à ampliação e transformação da saúde digital no Brasil.

### Limitações do estudo

O estudo foi limitado pelas poucas fontes de informações para desenvolver a revisão integrativa. Além disso, não foi disponibilizada linguagem para surdos e mudos, portanto a saúde digital inclusiva não foi atendida.

### Contribuições para as áreas das ciências e enfermagem

No que se refere às implicações ao avanço do conhecimento científico para a saúde e enfermagem, o aplicativo Criança e Cuidado, após ser validado, será disponibilizado para uso público, visando alcançar o máximo de escolas brasileiras e ser utilizado por enfermeiras no exercício da educação em saúde. Poderá ser oferecido, ainda, como instrumento de gestão nas Secretarias de Saúde e Educação.

## CONCLUSÕES

Foi desenvolvida inovação tecnológica no formato de aplicativo móvel sobre primeiros socorros a crianças para uso por profissionais da educação básica, o que vai possibilitar o registro dos acidentes ocorridos dentro do ambiente escolar. A ferramenta está estruturada em três *moblets,* a saber: Contatos importantes; Aprenda primeiros socorros; e Registre aqui o acidente. Ela possui recursos como vídeos, imagens e informações em texto organizado em tópicos.

O aplicativo é uma ferramenta educacional tecnológica que, após validação, possibilitará a ampla divulgação de informações essenciais para proteger a vida e saúde das crianças. Assim, representa uma iniciativa de enfrentamento das lesões não intencionais infantis, que figuram como importante problema de saúde pública no país e se enquadram no perfil de morbimortalidade infantil.
